# Genome-Wide Analysis of the YABBY Gene Family in Grapevine and Functional Characterization of *VvYABBY4*

**DOI:** 10.3389/fpls.2019.01207

**Published:** 2019-10-08

**Authors:** Songlin Zhang, Li Wang, Xiaomeng Sun, Yunduan Li, Jin Yao, Steve van Nocker, Xiping Wang

**Affiliations:** ^1^State Key Laboratory of Crop Stress Biology in Arid Areas, College of Horticulture, Northwest A&F University, Yangling, China; ^2^Key Laboratory of Horticultural Plant Biology and Germplasm Innovation in Northwest China, Ministry of Agriculture, Northwest A&F University, Yangling, China; ^3^College of Horticulture, Agricultural University of Hebei, Baoding, China; ^4^Department of Horticulture, Michigan State University, East Lansing, MI, United States

**Keywords:** grapevine, seedlessness, transcription factor, YABBY, gene function

## Abstract

Genes of the plant-specific YABBY transcription factor family have various roles, including lateral organ development, establishment of dorsoventral polarity, and response to abiotic stress. In this study, we carried out a genomic census of YABBY genes in grapevine (*Vitis vinifera*) and characterized their expression pattern during ovule development. We identified seven YABBY genes and classified them into five subfamilies, based on peptide sequence, similarity of exon–intron structure and composition of peptide sequence motifs. Analysis of YABBY gene expression in various grapevine structures and organs suggested that these genes function in diverse aspects of development and physiology. Analysis of expression during ovule development in four cultivars showed that one gene, *VvYABBY4*, was preferentially expressed during the period of ovule abortion in seedless cultivars. Transgenic expression of *VvYABBY4* in tomato conferred reduced plant stature, dark green leaves, elongated pistil, and reduced size of fruit and seeds. Reduced seed size was associated with smaller endosperm cells. Expression of *VvYABBY4* also affected expression of numerous tomato genes with presumed roles in seed development. These data suggest the potential for *VvYABBY4* to influence seed development in grapevine, which may impact seedless grape breeding.

## Introduction

The plant-specific YABBY transcription factors play important roles in the establishment of adaxial–abaxial polarity (Kumaran et al., 2002), development of lateral organs (Bowman et al., 1989), and stress response (Zhao et al., 2017). YABBY proteins comprise two highly conserved DNA-binding domains: a zinc finger-like domain (C2C2) and helix–loop–helix domain (YABBY) (Jang et al., 2004). The genome of *Arabidopsis thaliana* (Arabidopsis) encodes for six YABBY members, which have been classified into five subfamilies, designated FIL/YAB3, CRC, INO, YAB2, and YAB5. *FILAMENTOUS FLOWER* (*FIL*), *YAB3*, *YAB2*, and *YAB5* act redundantly to promote lateral organ development. *CRABS CLAW* (*CRC*) participates in establishing polarity of developing carpels and nectaries (Siegfried et al., 1999). *INNER NO OUTER* (*INO*) plays a seemingly distinct role to promote development of the ovule outer integument, the layer of cells surrounding the nucleus, into the seed coat (Villanueva et al., 1999; Bowman, 2000).

Based on their critical functions in development, the genomic organization and expression of YABBY genes have been studied widely in plants. In maize, there are 13 YABBY genes, and at least a subset of these is strongly expressed in the shoot meristem and leaf primordia, suggesting a function in organ formation and polarity (Sawa et al., 1999; Siegfried et al., 1999). Rice contains eight YABBY genes, including the well-studied *DROOPING LEAF* (*DL*) (Tanaka et al., 2017). In a loss of function *dl* mutant, carpels are transformed to stamens, and formation of leaf veins is defective (Yamada et al., 2004; Tanaka and Hirano, 2012). *DL* is orthologous with *CRC*, and other members of the *CRC* subfamily have been reported to be associated with polarity (Nagasawa et al., 2003; Ohmori et al., 2008). Tomato contains nine YABBY genes, and Cong found that *FASCIATED* (*SlYABBY2b*) mainly controls the formation of carpel during the flowering and fruit development of tomato (Cong et al., 2008). *SlYABBY1a* was highly expressed in the epidermis, and it is speculated that the *SlYABBY1a* gene may affect the development of distal pericarp cells and play an important role in the regulation of fruit ripening (Bartley and Ishida, 2003; Bartley and Ishida, 2007). Previously, Xiang et al. cloned two genes from the wild Chinese grapevine species, *Vitis pseudoreticulata*, and studied their function by heterologous expression in Arabidopsis. They found that the expression of *VpYABBY1* caused the loss of dorso-ventral polarity in the leaf blade, similar to previous observations for *FIL* and *YABBY3* (Siegfried et al., 1999; Xiang et al., 2013).

Most reports on YABBY genes have examined potential functions in symmetry of shoot apical and floral meristems in Arabidopsis (Siegfried et al., 1999; Kumaran et al., 2002). By contrast, there have been few previous investigations into the YABBY genes in common grapevine (*Vitis vinifera* L.). In this study, we investigated the genomic organization and expression of the YABBY gene family in grapevine, focusing on those genes that may have a role in seed development or abortion. Our results have potential significance for molecular breeding programs of seedless grapes.

## Materials and Methods

### Plant Material

Four grapevine cultivars, including two seedless cultivars, Flame Seedless (*V. vinifera*) and Thompson Seedless (*V. vinifera*), and two seeded cultivars, Red Globe (*V. vinifera*) and Kyoho (*V. vinifera* × *Vitis labrusca*), were used in this study. Plants were maintained in the grape germplasm resource collection of Northwest A&F University, Yangling, China (34°20′N108°24′E). Roots, stems, leaves, flowers (at full bloom stage), tendrils, and fruit (42 days postanthesis) were collected from 4-year-old “Red Globe” and “Thompson Seedless” plants. As noted previously (Li et al., 2017b), fertilized ovules in seedless cultivars typically abort at 30 to 40 days after full bloom (DAF). Consequently, ovules and seeds were dissected from developing fruit at 27 DAF, 30 DAF, 33 DAF, 36 DAF, 39 DAF, and 42 DAF. Samples were frozen immediately in liquid nitrogen and stored at −80°C for analysis of gene expression.

Genetically modified tomato (genotype micro-Tom) and the harvested seeds (T1) were cultivated in a greenhouse under 14-h light/10-h dark photoperiods, 25°C day/20°C night temperatures, and relative air humidity of 60%. Plant nutrient solution was applied once a week.

### Identification of YABBY Genes in the Grapevine Genome

The YABBY signature domain (PF04690) was identified with SMART (http://smart.embl-heidelberg.de) (Lu et al., 2017). The Hidden Markov Model (HMM) profile of the YABBY DNAbinding domain (PF04690) was obtained from the Pfam protein families database (http://pfam.xfam.org/) (Punta et al., 2012). Candidate genes were obtained from the 12 coverage assembly of the *V. vinifera* PN40024 genome (12×; http://www.genoscope.cns.fr) by using HMM online software (Eddy, 1998; Finn et al., 2015). Open-reading frames (ORFs) were obtained by ORF finder (http://www.ncbi.nlm.nih.gov/gorf/gorf.html). Detailed information, including gene ID, protein sequence, and coding sequence, was retrieved from the Grape Genome Database and NCBI GenBank. The number of amino acids and isoelectric point were obtained by ExPASy (http://web.expasy.org/protparam/).

### Multiple-Sequence Alignment and Phylogenetic Analyses

Multiple alignment of YABBY protein sequences from grapevine, Arabidopsis (Bowman, 2000), tomato (Huang et al., 2013), rice (Toriba et al., 2007), and maize (Juarez et al., 2004) were performed using ClustalX 2.1 (Thompson et al., 2002). Phylogenetic tree was constructed with the neighbor-joining (NJ) method using MEGA 5.0 (Stracke et al., 2001). Bootstrap analysis was performed using 1000 replicates with the following parameters: “p distance,” “complete deletion,” and “gap setting” (Sun et al., 2017).

### Distribution of Conserved Domains and Exon–Intron Structure Analysis of VvYABBY Genes

Conserved sequence motifs in YABBY genes were identified using MEME 4.11.2 (http://meme-suite.org/tools/meme)(Nakano et al., 2006); protein domains were identified using SMART (http://smart.embl-heidelberg.de)(Li et al., 2017a; Zhang et al., 2017). Exon–intron structures were determined according to the alignments of their transcribed sequences with corresponding genomic sequences, and the diagram was generated with the online Gene Structure Display Server (http://www.cbi.pku.edu.cn/resource/index.htm)(Wang et al., 2015).

### Determination of Chromosomal Localization and Synteny Analysis

Genomic positions of the YABBY genes were as previously annotated in the Grape Genome Database (12×; http://www.genoscope.cns.fr). Tandemly duplicated genes were defined as adjacent to homologous YABBY genes, with no more than one intervening gene (Holub, 2001). Syntenic blocks within the grape genome, as well as between grape and Arabidopsis, were identified from the Plant Genome Duplication Database (http://chibba.agtec.uga.edu/duplication) (Zhang et al., 2012b).

### Gene Expression Analysis

Total RNA was isolated from 100 to 200 mg of all the samples using an EZNA Plant RNA Kit (R6827-01; Omega Bio-Tek, Norcross, GA, USA), according to the manufacturer’s instructions. Agarose gel electrophoresis was used to check the integrity of RNA. Moreover, total RNA was quantified with Nanodrop8000 Spectrophotometer (Thermo Scientific, Waltham, MA, USA). One microgram of total RNA was used to synthesize first-strand cDNA using the Prime Script RT reagent Kit (TaKaRa Biotechnology, Dalian, China) in a final volume of 20 μl. The products were diluted sixfold for further experiments.

Quantitative real-time PCR analysis was performed with an IQ5 real-time PCR machine (Bio-Rad, Hercules, CA, USA). Each reaction was conducted in triplicate with a final volume of 20 μl, including 1.6-μl primer (1.0 μM), 1.0-μl cDNA template, 10.0-μl SYBR green (TaKaRa, Bio Inc.), and 7.4-μl sterile distilled H_2_O. The PCR amplification profile used was following a previous study (Wang et al., 2015). Relative expression levels were analyzed using IQ5 software and the normalized expression method (Guo et al., 2014). The sequence of the gene-specific primers used for each of the YABBY genes in grapevine, as well as two house-keeping *VvACTIN* (GenBank Accession number AY680701) and *VvEF1-α* (GenBank Accession number EC931777) gene, is listed in **Table S1**. The CT values of the *VvACTIN* and *VvEF1-α* were geometrically averaged as the new CT (internal control gene). The relative transcript level of a gene of interest is calculated as 2^−ΔΔCT^ [ΔΔCT = ΔCT (candidated sample) − ΔCT (control sample); ΔCT = CT (interesting gene) − CT (internal control gene)] (Guo et al., 2018).

### Subcellular Localization and Transactivation Assay of *VvYABBY4*

The coding region of *VvYABBY4* gene without terminator code was amplified and connected with pBI221–GFP vector. The recombinant plasmid pBI221–VvYABBY4–GFP and empty vector were transformed into *Agrobacterium tumefaciens* strain GV3101. The leaves of 5-week-old tobacco (*Nicotiana benthamiana*) were injected with bacteria solution. After 72-h treatment, leaves were observed under a Nikon A1R/A1 confocal microscope (Nikon, Tokyo, Japan). GFP was excited at 514 nm. DAPI was excited at 405 nm (Fang et al., 2016).

Yeast GAL4 system was used for transactivation assay. Since p53 and large T-antigen are known to interact in a yeast two-hybrid assay (Zhu, 1997; Li and Fields, 1993), it can be used as a positive control. A negative control should also be performed using pGBKT7-Lam (which encodes the Gal4 BD fused with lamin) and pGADT7-T. Then, select the colonies as the positive and negative control, respectively. These diploids are useful as reference strains for checking new batches of growth media (pGBKT7-53–positive control bait plasmid, pGBKT7-Lam–negative control bait plasmid, and pGADT7-T–positive control prey plasmid purchased from TAKARA company, 630489).The full-length ORF of VvYABBY4 was cloned into the pGBKT7 vector with Sma I and Sal I cleavage sites. The empty pGBKT7 vector and recombinant vector pGBKT7–VvYABBY4 were transformed into the Y2H yeast strain for the transcriptional activation analysis. These constructs were painted on the same SD/Trp−, SD/Trp−/X-α-gal or SD/Trp−/X-α-gal/AbA medium plates at 30°C for 3 to 4 days. (Reference Matchmaker™ Gold Yeast Two-Hybrid System User Manual; clontech, TAKARA).

### Molecular Cloning of the *VvYBBY4* Gene and Expression in Transgenic Tomato

The *VvYABBY4* cDNA was engineered into the pCAMBIA2300 vector under control of the CaMV 35S promoter, making use of the primers shown in **Table S2**. The resulting pCAMBIA2300–*VvYABBY4* plasmid was introduced into *Agrobacterium* strain GV3101. Transformation was carried out using the leaf disc method (Mccormick et al., 1986). Primers were designed according to the sequence of pCAMBIA2300 vectors and used for PCR identification of transgenic lines.

### Analysis of Transgenic Plants

Pollen viability and germination were evaluated in accordance with the method used in previous study (Zhang et al., 2012a; Pham et al., 2015). Pollen morphology was observed under a scanning electron microscope (SEM). Sample preparation for SEM was carried out according to previous study (Xiang et al., 2013). Cell measurements were performed using Image-pro software.

## Results

### Identification of YABBY Gene Family Members in *V. Vinifera*

Based on the presence of an ORF encoding a single, conserved YABBY domain, we identified seven YABBY genes in a draft genome sequence of grapevine. We designated these genes *V. vinifera YABBY1*–*YABBY7* according to their consecutive chromosomal positions. The parameters used to describe the VvYABBY proteins are listed in **Table 1** and included gene locus identifiers, gene accession numbers, chromosomal position, pI, and length of the coding sequence. The deduced length of VvYABBY proteins ranged from a minimum of 160 amino acids (VvYABBY1) to a maximum of 211 residues (VvYABBY3), whereas the isoelectric point (pI) values ranged from 7.03 (VvYABBY2) to 8.98 (VvYABBY1). This variability suggests that different VvYABBY proteins might play a part in different microenvironments.

**Table 1:** Information of grape YABBY genes/proteins.

Gene locus ID	Gene name	Accession no.	Chromosome	Start	End	CDS (bp)	ORF (aa)	Isoelectric point (pI)
GSVIVT01012246001	VvYABBY1	CBI27316	chr1	237202	239435	483	160	8.98
GSVIVT01013778001	VvYABBY2	CBI28560	chr1	7691531	7692677	531	176	7.03
GSVIVT01001269001	VvYABBY3	CBI31883	chr2	4861965	4864774	636	211	7.71
GSVIVT01037533001	VvYABBY4	CBI24407	chr6	11951270	11958015	552	183	8.38
GSVIVT01022586001	VvYABBY5	CBI39135	chr8	5502500	5509539	555	184	8.68
GSVIVT01015567001	VvYABBY6	CBI28225	chr11	5013887	5017668	558	185	8.43
GSVIVT01027648001	VvYABBY7	CBI38884	chr15	14674818	14677943	633	210	8.57

CDS, coding sequence; ORF, open-reading frame; Chr, chromosome.

### Multiple Sequence Alignment and Structural Analysis of YABBY Genes in Grape

**Figure 1:**
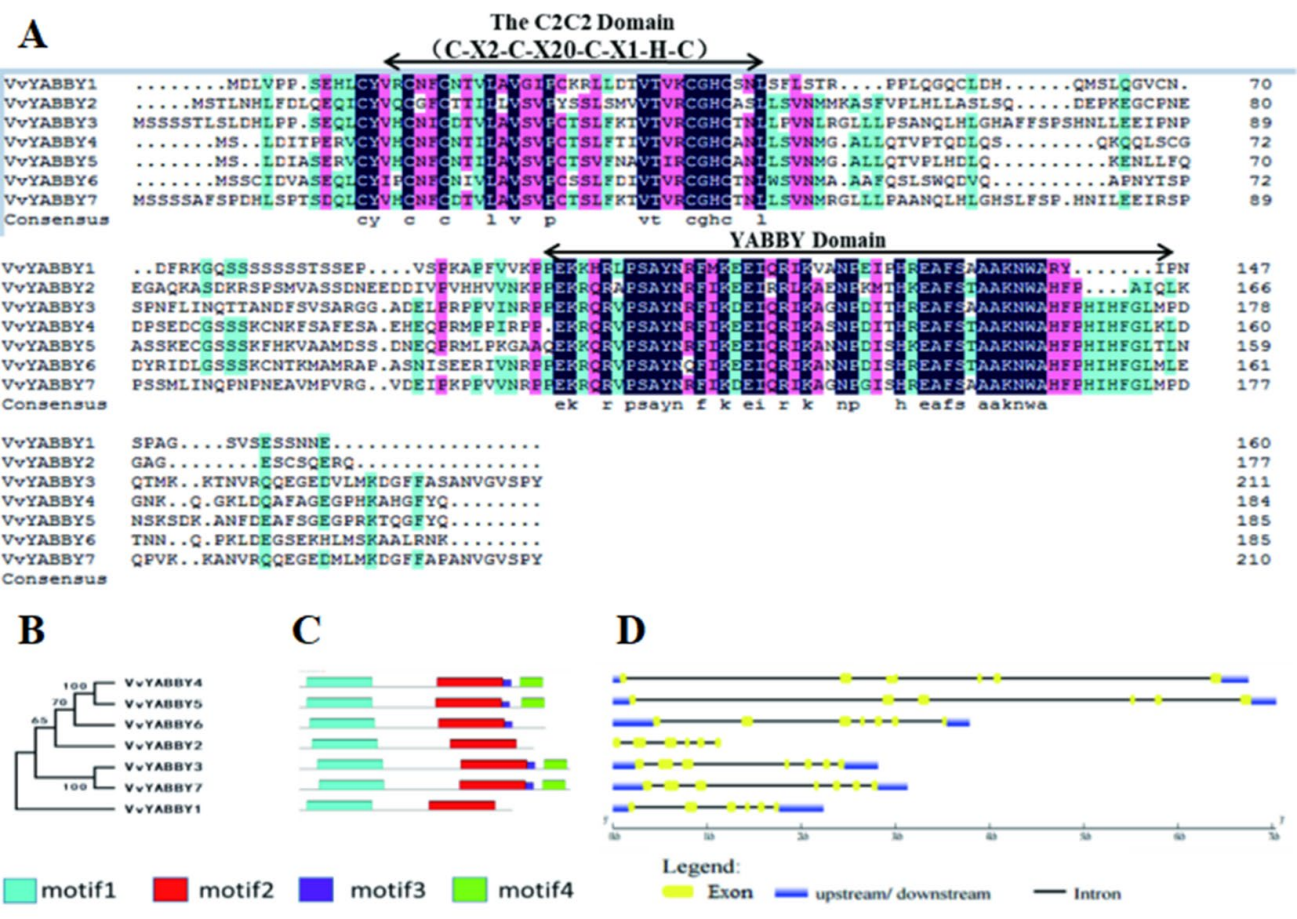
YABBY genes in grapevine. **(A)** Alignment of open-reading frame translations from the *Vitis vinifera* YABBY genes. The conserved C_2_C_2_ and YABBY domains are marked. **(B)** Phylogenetic relationships among the ORF translations of the seven grapevine YABBY genes. Numbers near the nodes represent bootstrap values. **(C)** Motif analysis of grapevine YABBY ORF translations. Motifs were designated as 1–4 and distinguished by different colors. **(D)** Exon–intron structure of grapevine YABBY genes. Exons are indicated as yellow boxes. Black lines linking two exons represent introns.

A sequence alignment of the YABBY proteins is shown in **Figure 1A**. The ∼60-amino acid YABBY domain is situated near the carboxyl terminus, similar to YABBY proteins in other plants where characterized. All grape YABBYs also contain a highly conserved C_2_C_2_ zinc finger domain near the amino terminus. Within this domain, the spatial configuration of cysteine (C) and histidine (H) residues directly involved with Zn^2+^ binding are conserved (C-X2-C-X20-C-X1-HC). Analysis of additional conserved segments within the VvYABBY1–VvYABBY7 protein sequences revealed a short domain bordering the YABBY domain in *VvYABBY3*, *4*, *5*, *6*, and *7*, and a carboxyl-terminal domain in *VvYABBY3*, *4*, *5*, and *7* (**Figure 1B**). Consistent with the similarity in domain composition, a phylogeny based on peptide sequence revealed a close relationship between *VvYABBY4* and *VvYABBY5*, and *VvYABBY3* and *VvYABBY7* (**Figures 1B, C**). This was further supported by analysis of the exon–intron structures of the seven genes, which identified similar exon partitions between *VvYABBY4* and *VvYABBY5*, and *VvYABBY3* and *VvYABBY7* (**Figure 1D**).

### Phylogenetic and Synteny Analyses of YABBY Genes

**Figure 2:**
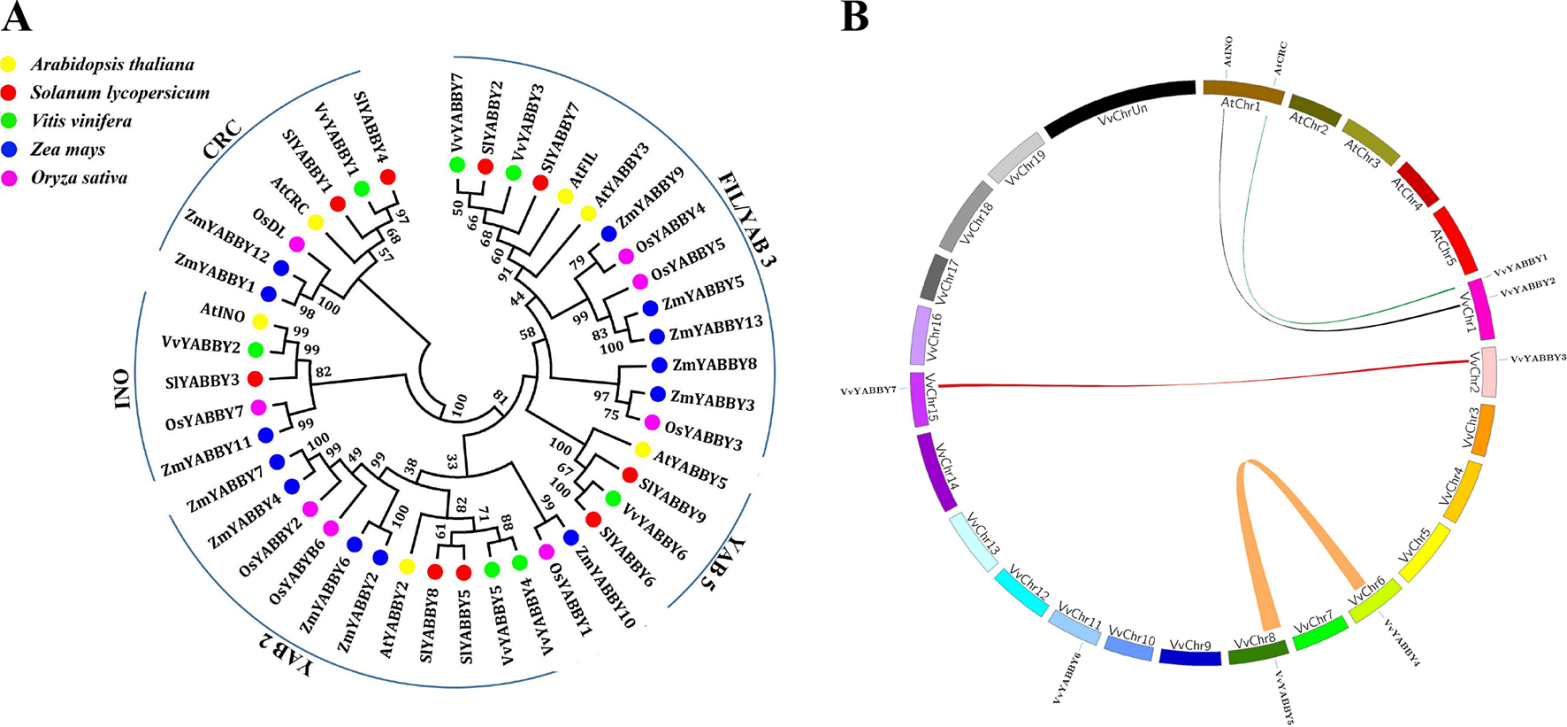
Phylogenetic relationships and synteny analysis of grapevine YABBY genes. **(A)** Phylogenetic analysis of YABBY genes from grapevine and other species. Yellow circles represent *Arabidopsis thaliana*, red circles represent *Solanum lycopersicum*, green circles represent *Vitis vinifera*. Blue and purple circles denote *Zea mays* and *Oryza sativa*, respectively. Numbers at the nodes indicate bootstrap values; values lower than 30% are not shown. **(B)** Synteny analysis of YABBY genes between grape and *Arabidopsis*. Colored bars linking two chromosomal parts represent syntenic regions. Chr, chromosomes.

To better understand the evolutionary relationships among YABBY genes, a phylogenetic tree was constructed with a total of 43 YABBY genes from different species (**Figure 2A**). As important model plants, the functions of Arabidopsis and tomato YABBY genes have been well discussed, allowing inference of function for phylogenetically related proteins from grape. This analysis divided the grapevine YABBY genes into five well-defined clades or subfamilies, consistent with previous analysis in Arabidopsis and tomato: 1) FIL/YAB3, 2) YAB2, 3) YAB5, 4) INO, and 5) CRC. It is evident that YABBY genes of the monocotyledon (rice and maize) clustered together. As expected, grape YABBY genes showed a closer relationship to dicotyledons (Arabidopsis and tomato). In clade YAB5, no genes of monocotyledon species was included. In addition, as a distinct branch, *ZmYABBY10* and *OsYABBY1* are highly homologous but not homologous with any dicotyledonous plants. It shows that the YABBY gene is not conservative in the evolution of species, and they have diversified during their evolution. The clear orthology observed within each of these clades among the five species suggests that these subfamilies were distinguished prior to the evolutionary separation of these plants.

As shown in **Figure 2B**, two pairs of grape YABBY genes (*VvYABBY3/VvYABBY7* and *VvYABBY4/VvYABBY5*) are associated with segmental duplication (**Table S3**). Moreover, studies of syntenic relationships often allow transferring available genomic information from a model species to a less-studied species (Lyons et al., 2008). Some of the predicted YABBY domains from grapevine and Arabidopsis showed pairwise relationships, such as those between *VvYABBY1*–*AtCRC* and *VvYABBY2*–*AtINO* (**Table S4**), and this is consistent with the grouping in **Figure 2A**. This suggested that they came from a common ancestor and duplicated prior to the divergence of grape and Arabidopsis.

### Expression Profiles of YABBY Genes in Diverse Structures

**Figure 3:**
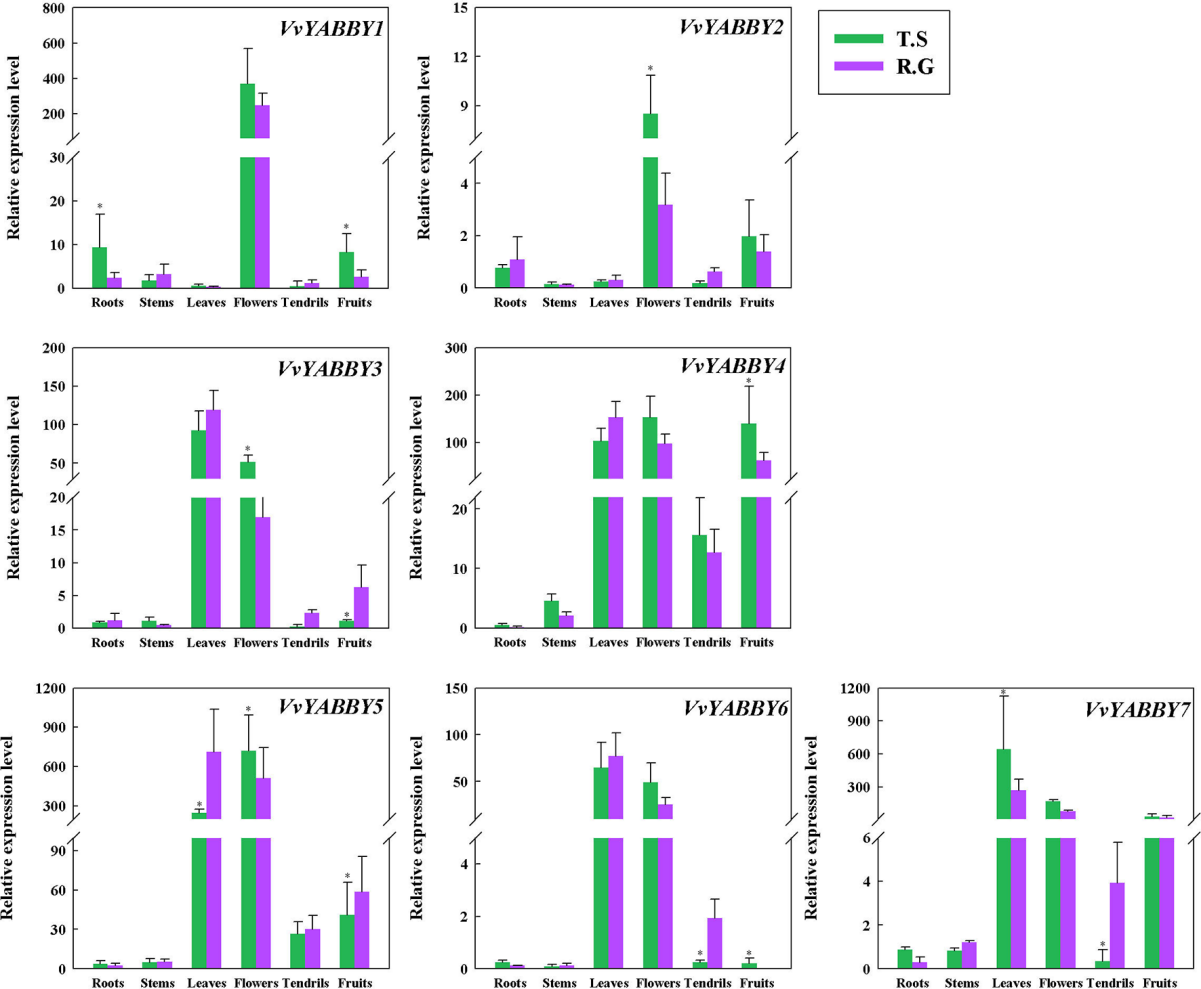
Analysis of grape YABBY genes expression in various grapevine structures by quantitative PCR. “T.S.” stand for “Thompson Seedless” and “R.G.” represent “Red Globe.” Using *VvACTIN* and *VvEF1*-α genes followed by geometric averaging for determining a normalization factor. The means ± SD of three biological replicates are presented. Asterisks represent statistical significance (*P < 0.05, one-way ANOVA).

As the first step to define function of the grapevine YABBY genes, we evaluated their expression levels in stems, young leaves, roots, flowers, tendrils, and fruit. In the seeded cultivar “Red Globe” and seedless cultivar “Thompson Seedless”,: all of the genes showed relatively high expression in the flower (**Figure 3**). *VvYABBY3*, *4*, *5*, *6*, and *7* were also relatively highly expressed in the leaf. In addition, *VvYABBY4*, *5*, and *7* showed relatively high expression in fruit. *VvYABBY1*, *3*, and *7* were expressed relatively strongly in root. Comparing these results with our phylogenetic and synteny analyses, it is interesting that some homologous genes (*VvYABBY3* and *VvYABBY7*, for example) displayed a similar expression pattern in all structures.

### Expression Patterns of Grape YABBY Genes During Ovule Development Among Seeded and Seedless Cultivars

**Figure 4:**
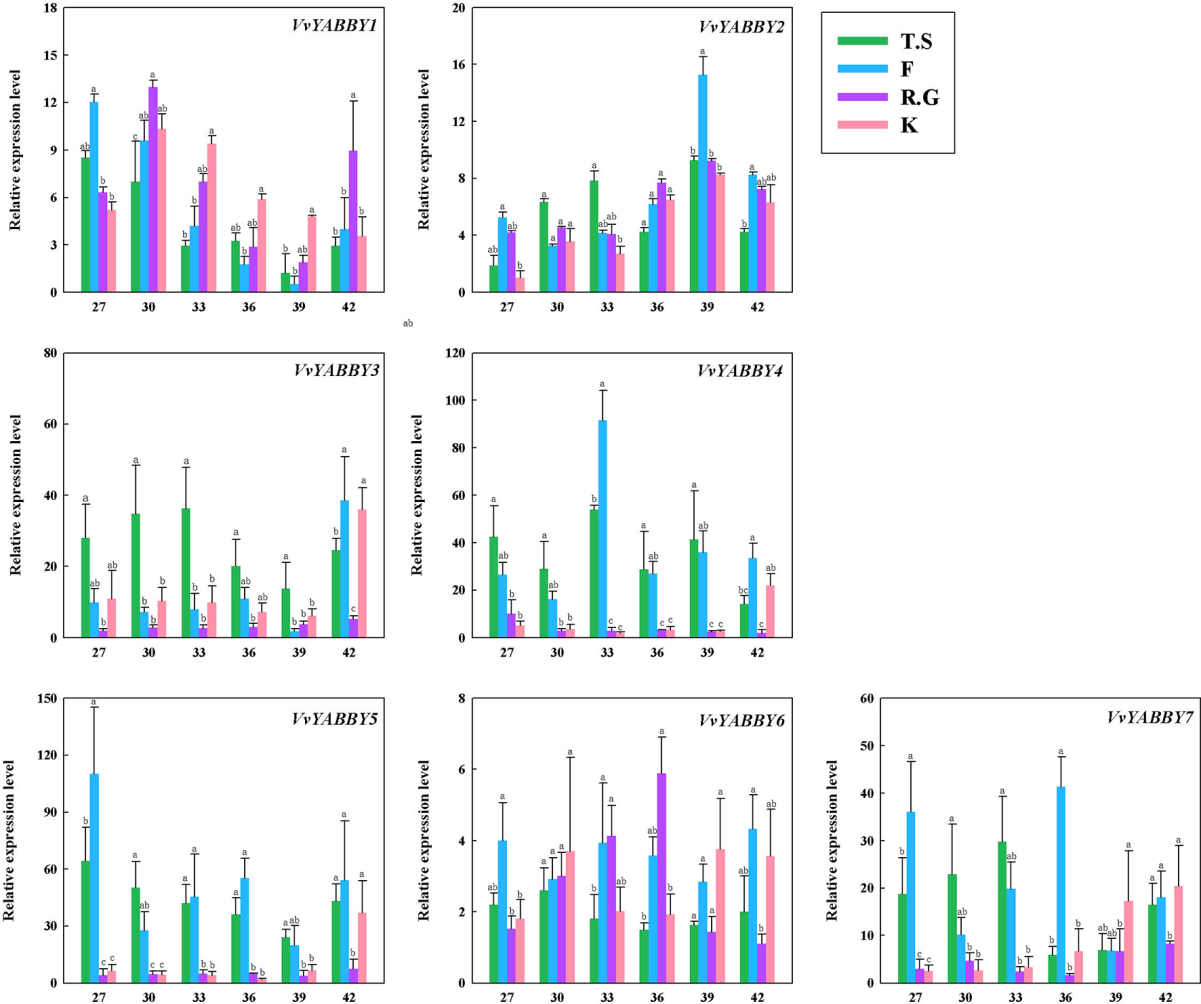
Expression pattern of grape YABBY genes in different stages of ovule development in four grapevine cultivars. Seedless cultivars: Thompson Seedless (T.S) and Flame Seedless (F). Seeded cultivars: Red Globe (R.G.) and Kyoho (K). The value of the x - axis represents the days after full bloom. Using *VvACTIN* and *VvEF1*-α genes followed by geometric averaging for determining a normalization factor. The means ± SD of three biological replicates are presented. Values with different lowercase superscripts mean significant difference (*P* < 0.05).

The expression of YABBY genes in four grapevine cultivars, including two seedless cultivars, Flame Seedless (*V. vinifera*) and Thompson Seedless (*V. vinifera*), and two seeded grapevines, Red Globe (*V. vinifera*) and Kyoho (*V. vinifera* × *V. labrusca*), are shown in **Figure 4**. Some of the genes, like *VvYABBY1*, *2*, and *6,* exhibited no striking differences in expression pattern during ovule development between seedless and seeded grape cultivars. On closer inspection, however, it becomes clear that *VvYABBY3*/*VvYABBY7* and *VvYABBY4/VvYABBY5* also had the same pattern of expression during the development of ovule. In addition, we can conclude that YABBY genes participate in the development of ovules, because all of them were expressed during ovule growth.

It is noteworthy that *VvYABBY4* and *VvYABBY5* were expressed to relatively high levels in the two seedless cultivars. According to previous studies, ovule abortion usually occurs between 30 and 40 DAF (Li et al., 2017b). During this period, the level of *VvYABBY4* gene expression increased with the beginning of ovule abortion and then decreased. However, the expression level of *VvYABBY5* was more stable compared with *VvYABBY4*. It suggests that ovule abortion begins with the up-regulation of *VvYABBY4* gene. This hypothesis, though, needs further proof.

### *VvYABBY4* Is Positioned in the Nucleus and Functions as a Transcriptional Activator

**Figure 5:**
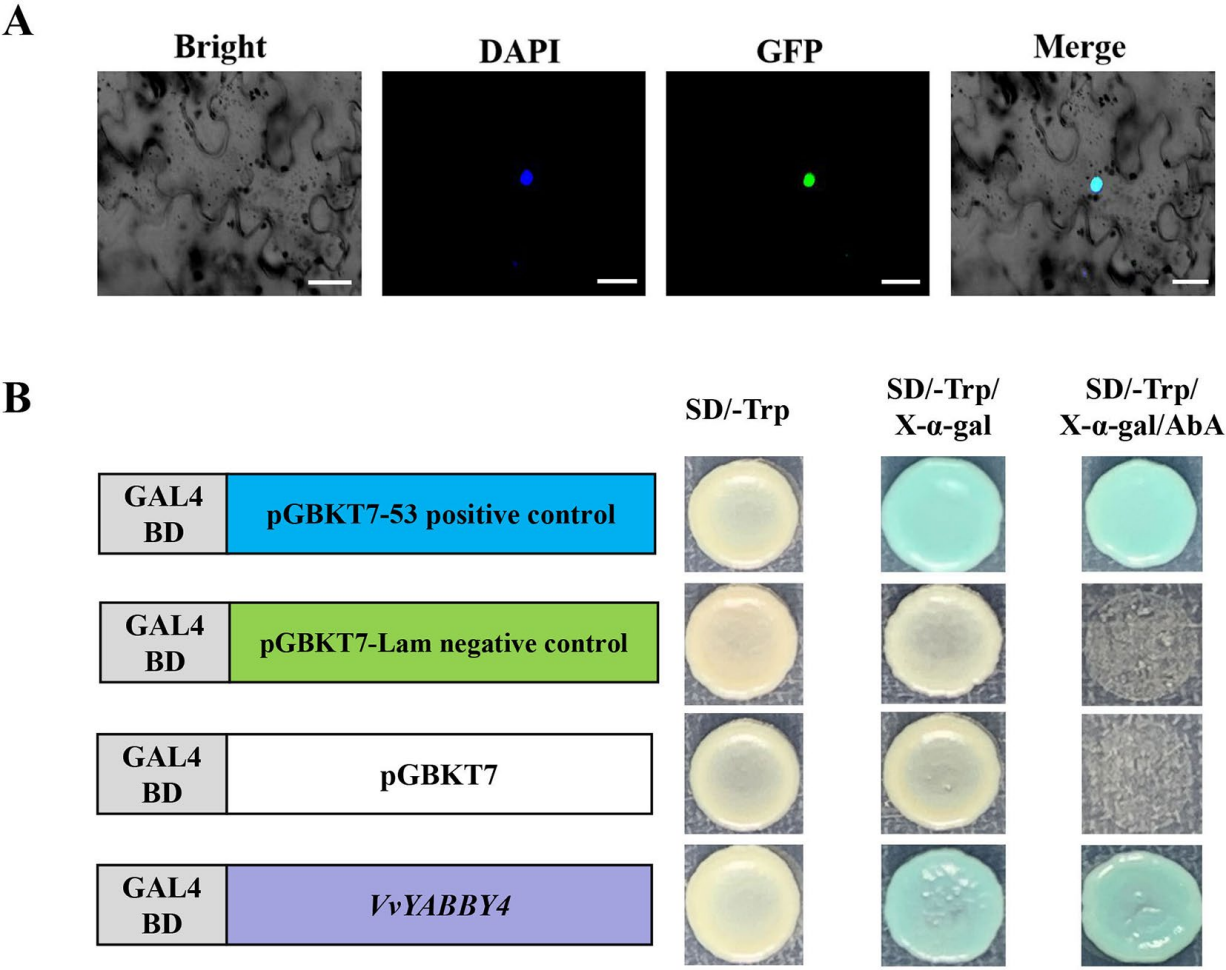
Subcellular localization and transactivation analysis of *VvYABBY4*. **(A)** Subcellular localization of the 35S::*VvYABBY4*–eGFP fusion protein in *Nicotiana benthamiana* leaves. Scale bar: 20 μm. **(B)**. Transactivation activity analysis of full length *VvYABBY4* in yeast.

Recombinant plasmid pBI221–VvYABBY4–GFP was transfected into tobacco leaf. In the case of DAPI, the fluorescence signal was alone detected in the nucleus; therefore, the pBI221–VvYABBY4–GFP fusion protein was localized to the nucleus (**Figure 5A**). To analyze the transcriptional activation of *VvYABBY4*, full-length *VvYABBY4* was inserted into the pGBKT7 vector. Results showed that yeast transformed with pGBKT7-VvYABBY4 had activation ability and presented β-galactosidase activity (**Figure 5B**). These results confirm that VvYABBY4 is an active transcription activator which localized to the nucleus.

### Characterization of the Tomato Expressing *VvYABBY4*

**Figure 6:**
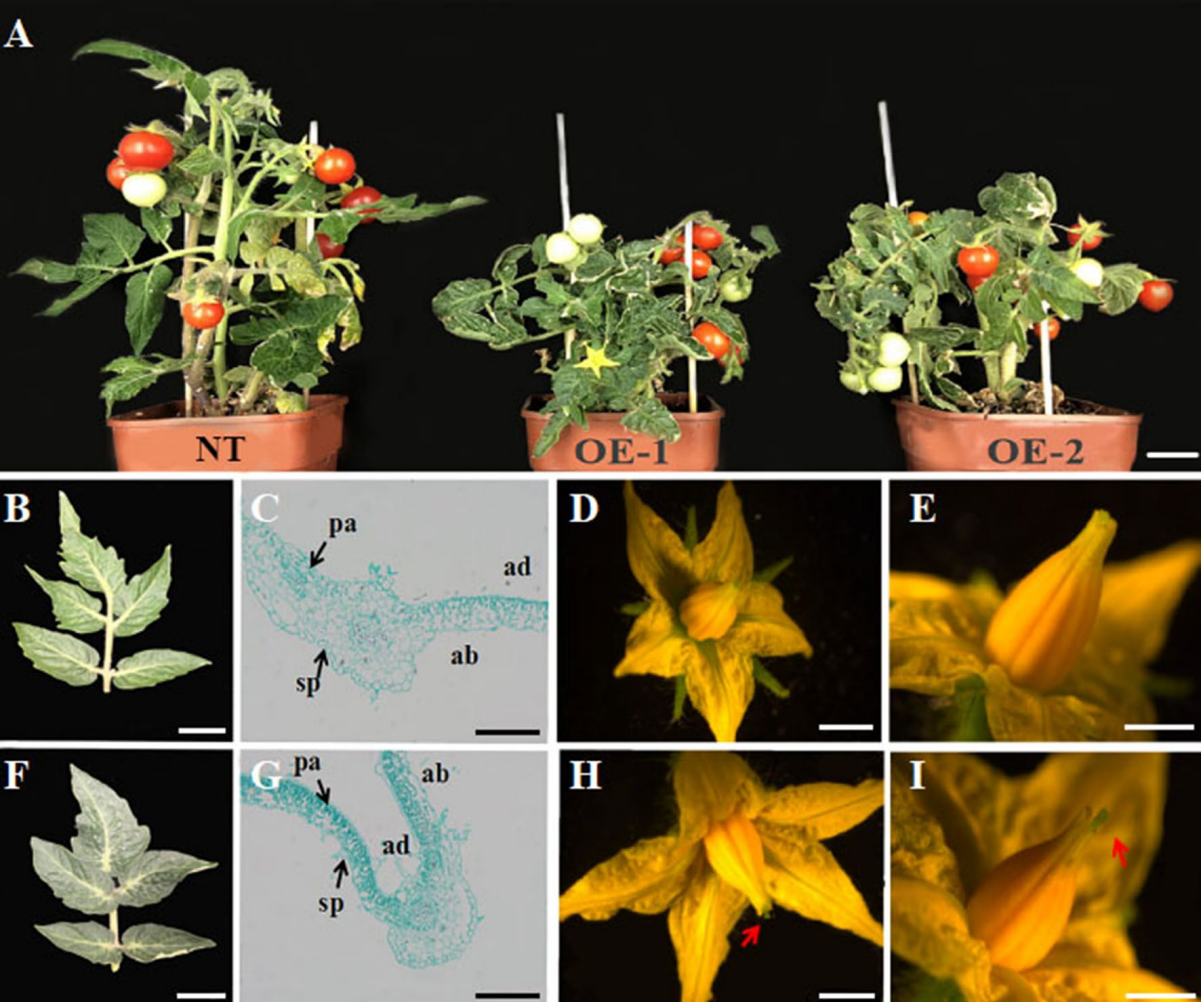
Phenotypic analysis of effects of *VvYABBY4* expression in transgenic tomato. **(A)** Observation of non-transgenic control plant (NT; left) and representatives of two *VvYABBY4* expressing transgenic lines (OE-1 and OE-2). **(B)** Leaf from non-transgenic plant. **(C)**. Longitudinal sections of non-transgenic plant leaf. **(D)** Flower from non-transgenic plant. **(E)**. High magnification of the non-transgenic plant pistil. **(F**–**I)** The observation of *VvYABBY4* transgenic plant corresponding to **(B**, **C**, **D**, **E)**, respectively. The red arrow indicates the protruding part of the pistil. Bars:**(A)**, 1 cm **(B**, **D**, **F**, **H)**, 10 mm **(C**, **G)**, 200 μm **(E**, **I)**, 1 mm.

To further define a functional role for the grapevine YABBY genes in development, we engineered and characterized transgenic tomato constitutively expressing *VvYABBY4*. PCR identification showed that the *VvYABBY4* gene has been successfully transformed in the tomato (**Figure S1**). Compared with non-transgenic plants, transgenic lines were shorter and displayed reduced apical dominance (**Figure 6A**). The leaves of transgenic lines were also darker green compared with the control (**Figures 6B, F**). Microscopic observation revealed that leaf mesophyll cells of transgenic plants were present at a higher density, with the increased concentration of chloroplasts potentially contributing to the dark green color. Moreover, mesophyll cells of transgenic plants displayed significantly enhanced polarity (**Figures 6C, G**). In the adaxial region, long square columnar palisade tissue cells were closely arranged, with narrow intercellular space. In contrast, in the abaxial region, the spongy tissue cells displayed a spherical shape. In addition, a closer view of the transgenic leaves revealed obvious long hair on the surface, which rarely appeared on leaves of non-transgenic control plants.

Finally, 35S::*VvYABBY4* transgenic plants had larger flowers (**Figures 6D, H**), and the pistil was longer than non-transgenic control plants at anthesis (**Figures 6E, I**). Besides that, there were no obvious difference between transgenic and control plants in other respects, such as bud, sepal, or stamen development.

### Pollen Viability, Germination Percentage, and Pollen Morphology

**Figure 7:**
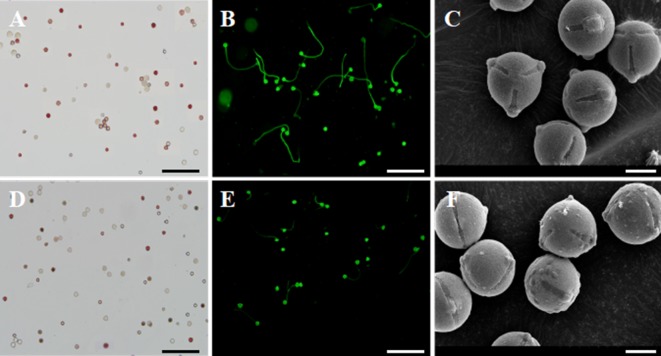
Experimentation of pollen energy, pollen germination and pollen grains. **(A**, **D)**. Pollen viability of non-transgenic control and *35S::VvYABBY4* transgenic plant using TTC staining 2 days before full-bloom respectively. **(B**, **E)**. Pollen germination of non-transgenic control and *35S::VvYABBY4* transgenic plant using aniline blue staining respectively. **(C**, **F)**. Observation of non-transgenic control and *35S::VvYABBY4* transgenic plant pollen under scanning electron microscope (SEM). Bars: **(A**, **B**, **D**, **E)**, 100 μm. **(C**, **F)**, 30 μm.

Pollen viability in transgenic plants was similar (∼85%) to non-transgenic plants, as evaluated using TTC staining (**Figures 7A, D**). In addition, transgenic and non-transgenic plants exhibited similar pollen size and shape. However, we found that the mature pollen grains from transgenic plants exhibited irregularly shaped protuberances attached to the exine (**Figure 7F**). In contrast, the pollen of non-transgenic plants appeared glabrous (**Figure 7C**). We collected pollen at the same time from transgenic and control plants and cultured the pollen under identical conditions. As shown in **Figures 7B, E**, the germination of pollen from 35S::*VvYABBY4* plants was slower than the control.

### Effect of *VvYABBY4* Expression on Transgenic Tomato Fruit and Seed Development and Morphology

**Figure 8:**
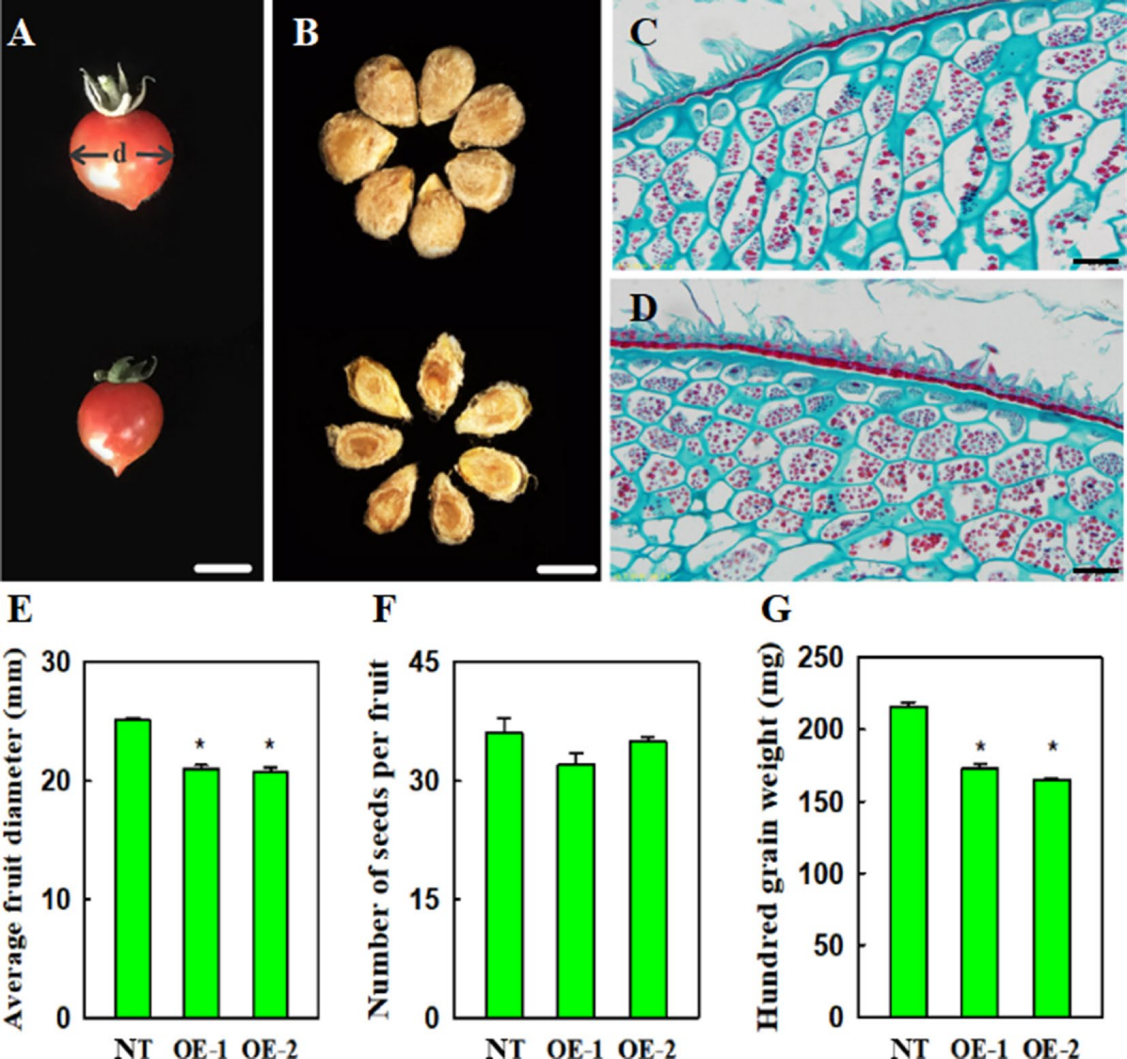
Effect of *VvYABBY4* expression on tomato fruit and seed development and morphology. **(A)** Fruit from non-transgenic control and transgenic plant (from top to bottom), “d” represents diameter. **(B)** Seeds from non-transgenic control and transgenic plant (from top to bottom).**(C)** and **(D)**. Histological observation of non-transgenic control and over-expression of *VvYABBY4* plants seeds. **(E)** Average fruit diameters of non-transgenic control (NT) and over-expression of *VvYABBY4* lines (OE-1 and OE-2). **(F)** Number of seeds per fruit in non-transgenic control (NT) and over-expression of *VvYABBY4* lines (OE-1 and OE-2). **(G)** Hundred grain weight of non-transgenic control (NT) and over-expression of *VvYABBY4* lines (OE-1 and OE-2). Values in **(E**-**G)** are given as means ± SEs, *P < 0.05, compared with the non-transgenic control. Three independent repeats were set with five fruits in each group and calculated the average of the three sets of data, total 15 fruits were used in one line test. The diameter of each single fruit was measured three times. Bars: **(A**, **B)**, 1 mm **(C**, **D)**, 20 μm.

Compared with non-transgenic control plants, transgenic plants, constitutively expressing *VvYABBY4*, exhibited slightly reduced fruit size, approximately 90.3% that of the control (**Figures 8A,E**). Relative to the seeds of non-transgenic plants, which were plump and wide, the seeds of transgenic lines were significantly smaller (**Figure 8B**). The hundred grain weight of seed from transgenic plants was reduced to 78.2% that of the controls (**Figure 8G**). However, the number of seeds per fruit was similar to controls (**Figure 8F**).

To obtain clues about the potential influence of *VvYABBY4* on seed development, we observed the seeds during period of mature green using microscopy (**Figures 8C, D**). Histological longitudinal section of transgenic plants seeds showed that endosperm cells were more closely arranged, and were narrower than those of nontransgenic plants. A potential explanation is that the development of the endosperm cells limits the seed size.

### Effect of *VvYABBY4* Expression in Transgenic Tomato on Genes Related to Seed and Fruit Development

**Figure 9:**
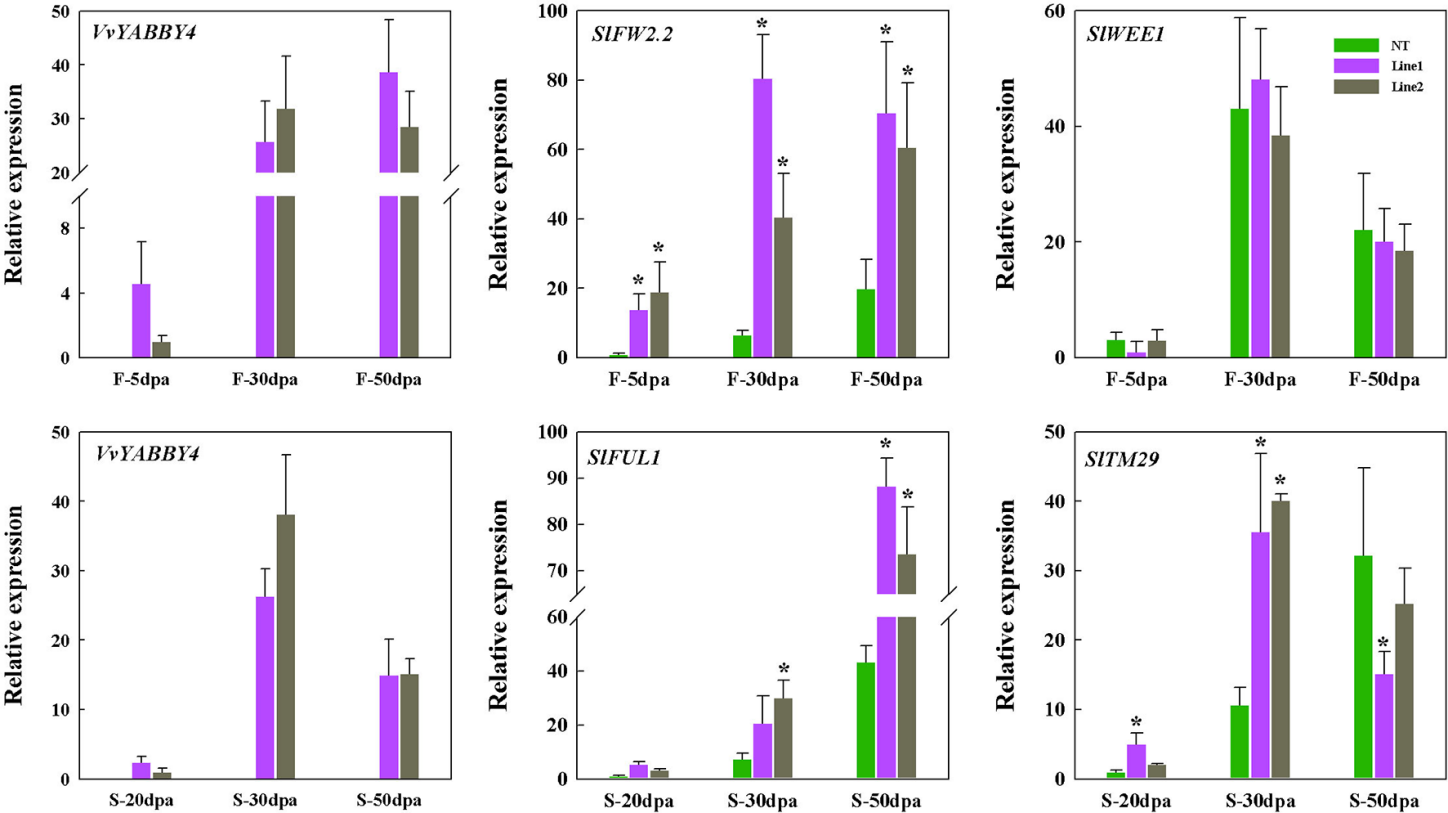
Expression of tomato genes linked with seed and fruit growth in *VvYABBY4* transgenic plants. Two independent lines (line 1 and line 2) were evaluated. Relative transcript accumulation of related genes in tomato fruits and seeds at different fruit development stages. Transcripts were normalized to the expression of the *SlACTIN1* gene. dpa, days postanthesis; F, fruit; S, seed; The means ± SD of three biological replicates are presented. Asterisks represent statistical significance (*P < 0.05, one-way ANOVA).

To index growth and development of the transgenic tomato seed and fruit at the molecular level, we analyzed the expression of four previously characterized tomato genes involved in seed or fruit growth and development. Tomato *FW2.2* encodes an unknown functional transmembrane protein and interacts with CKII kinase to form a cell cycle regulatory complex that inhibits cell division and participates in cell cycle signaling pathways (Mei and Simmons, 2011; Chakrabarti et al., 2013). Tomato *WEE1* participates in the control of cell size during fruit development through the negative regulation of CDK activity, resulted in a changes of plant and fruit size (Nathalie et al., 2004; Nathalie et al., 2007). As shown in **Figure 9**, the expression patterns of *SlWEE1* in transgenic plants are different from *SlFW2.2*. At all the stages of fruit development, the expression of *SlFW2.2* continues to be highly expressed compared with the control. However, the expression of *SlWEE1* has no obvious difference compared with non-transgenic plant during fruit development. This result indicated that the reduced fruit size of transgenic plant was not caused by the endoreduplication which was regulated by *SlWEE1*, but by the high expression of *SlFW2.2* caused by *VvYABBY4*, thus negatively regulating the fruit size.

As for seed size, we selected two genes that are involved in the development of tomato seeds. Tomato *TM29* functions in floral meristem, flower, and seed development (Sutherland et al., 2002). Tomato *FUL1* (*SlFUL1*, formerly known as *TDR4*) is expressed during ripening (Busi et al., 2003), where it interacts with *RIN* to mediate ethylene-independent aspects of fruit and seed ripening (Bemer et al., 2012). The expression of these two genes was assessed at three different seed developmental stages. As shown in **Figure 9**, at each stage, the expression of the *SlFUL1* in transgenic plants was higher than the control. *SlTM29* is highly expressed in the seeds of 30 days postanthesis compared with the controls, and the amount of expression dropped with the maturity of seeds. It has been reported that high expression of *SlFUL1* produced a smaller and reduced number of tomato seeds (Lozano et al., 1998; Czerednik et al., 2015) and decreased the expression of *SlTM29* conferred seedlessness in tomato (Sutherland et al., 2002). Our results are consistent with these observations and suggest that *VvYABBY4* gene may affect the expression of genes associated with seed growth to co-regulate seed development.

## Discussion

The YABBY family of plant-specific transcription factor plays an important role in abaxial–adaxial polarity and development of lateral organs. In addition, YABBY genes have been implicated as important components of plant response to abiotic stress. In this study, we identified seven YABBY genes in grapevine. Genomic studies have documented the existence of six YABBY members in Arabidopsis, nine in tomato, eight in rice, 17 in soybean, and 13 in maize. The relatively few YABBY genes found in grapevine could reflect the lack of expansion of this family during evolution.

To understand grape YABBY evolutionary relationships, we constructed a phylogenetic tree, including YABBY proteins from five species, including monocotyledons and dicotyledons. In all these plants, YABBY members were separated into five subfamilies. Genes in the same subfamily, such as *VvYABBY3* and *VvYABBY7*, or *VvYABBY4* and *VvYABBY5*, show similar motif and exon–intron structure. This apparent recent evolution implies that these gene pairs may perform a similar function. On the other hand, evolutionary differentiation of gene structure implies distinction of gene function(Wan et al., 2018). For example, in Arabidopsis, *CRC* is involved in the formation of nectaries and carpels, but *INO* is related to the outer integument development (Bowman, 2000).

Gene duplication and divergence events have been suggested to be the main contributors to evolutionary momentum (Ohno et al., 2010). We did not find any tandemly repeated gene events for the YABBY family. However, we identified two pairs of segmental duplications. Due to the genome of grapevine that originated from a hexaploidization (Jaillon et al., 2007), several synteny blocks would be expected to exist in this genome. Segmental duplicates may be more often retained due to subfunctionalization, without increasing the likelihood of gene rearrangement (Lynch and Conery, 2000; Lynch and Conery, 2003). Meanwhile, a total of two pairs of homologous genes were identified between grape and *Arabidopsis*, suggesting that they were derived from the same ancestor gene and became distinguished prior to species differentiation.

The function of Arabidopsis YABBY genes is related to development of abaxial tissues in lateral organs. For example, *FIL*, *YAB2*, and *YAB3* are expressed in cotyledons, leaves, flowers, and other lateral organs, whereas *CRC* is expressed in carpels. Similarly, YABBY genes in grapevine are widely expressed in many structures. Specifically, the relative expression of grape YABBY genes in floral organs is more obvious, suggesting that these YABBY genes are involved in the development of the flower. The observation that YABBY genes in the same subfamily showed similar patterns of expression, such as *VvYABBY3* and *VvYABBY7*, suggests that they perform similar functions. In our study, to complement previous research, we focused on identifying genes involved in seed development, as well as ovule abortion. Thus, we documented the expression of the seven genes in different stages of ovule development, in two seeded (Red Globe, Kyoho) and two seedless (Thompson Seedless, Flame Seedless) cultivars.

Quantitative results indicate that the expression of *VvYABBY4* and *VvYABBY5* showed significant differences between seeded and seedless grape varieties, this is also consistent with the published transcriptome data (**Table S5**) (Wang et al., 2016). And it is certain that due to different grape materials and sampling periods, there are some differences between us, just like in his results, there was no expression of *VvYABBY1* or *2* during the three stages. However, *VvYABBY3*, *4*, *5*, and *7* are significantly differentially expressed in seedless and seedless grapes. And these results are consistent with each other. Recent reports indicated that the key period of grape ovule abortion falls between 30 and 40 DAF (Wang et al., 2016; Li et al., 2017b). During this time, the expression level of *VvYABBY5* was not influenced by ovule abortion, although its expression in seedless grape varieties is excellent. By contrast, the level of *VvYABBY4* expression increased with the beginning of ovule abortion and then decreased with the end of procedure, and so we speculate that both *VvYABBY4* and *VvYABBY5* play a role in seed development, but that *VvYABBY4* plays an additional role in seed abortion.

It is worth noting that considering the sequence divergencies in four varieties (Genova et al., 2014), we cloned these seven YABBY genes, and the results showed that there was no difference in *VvYABBY1*, *2*, *3*, *4*, *6* sequences among the four cultivars (**Figure S2**). In addition, two points were found to be different from the reference genome in *VvYABBY5*, but they were exactly the same among the four species, which we doubt the accuracy of the reference genome at this site. The sequence alignment of YABBY7 in four varieties shows that YABBY7 has the same sequence in Kyoho and Pinot noir (reference genome), but two points are different from the other three varieties. So we designed the quantitative primer based on the sequencing results.

As for housekeeping genes in grape, grape berries or seeds undergo significant metabolic changes throughout their development, orchestrated in part by the up- and down-regulation of transcripts (Coombe, 1992). The ability to identify transcripts that are resistant to growth fluctuations or stresses is challenging, so to date, no single candidate housekeeping genes has been shown to be universally acceptable. In 2006, Reid demonstrated that the most stable reference genes: actin, GAPDH, EF1-α, and SAND ranked among the top genes when data from three independent statistical approaches were evaluated during grape development (Gonçalves et al., 2005; Reid et al., 2006). For more accurate normalization, use of at least two reference genes followed by geometric averaging is recommended for determining a normalization factor (Vandesompele et al., 2002; Reid et al., 2006). Therefore, two reference genes, *VvACTIN* and *VvEF1*-α, were used in our study.

Moreover, we carried out a preliminary exploration of the function of the *VvYABBY4* gene. The reduced apical dominance of the transgenic tomato plants is difficult to explain; however, it has been shown that overexpression of FW2.2 will dwarf corn plants (Mei et al., 2010), so we speculated that the overexpression of *VvYABBY4* led to the increase of FW2.2 expression, which inhibited the growth of the plant, given the presumed function of the YABBY genes. The more tightly packed arrangement of palisade cells in the 35S:*VvYABBY4* plant may lead to the apparent deeper green color. The surface of pollen grains showed irregular bulges, but this does not seem to affect normal pollination and fertilization. In addition, as shown in **Figure 2A**, *VvYABBY4* belong to the YAB2 subfamily, which is known to contribute to the development of lateral organs. Interestingly, we found that heterologous expression of *VvYABBY4* in tomato negatively impacts seed size, suggesting it may participate in seed development in grapevine. Similarly, the function of the rice YAB2-clade gene *DROOPING LEAF* is unrelated to lateral organ polarity (Jang et al., 2004; Yamada et al., 2004). Besides that, ectopic expression of *VpYABBY2* in Arabidopsis did not lead to any alteration in polarity. Rather it may play a specific role in carpel development and grape berry morphogenesis (Xiang et al., 2013). From these perspectives, we can conclude that during evolution of angiosperms, the functions of YABBY genes became increasingly diversified.

In addition, it was reported that seed size in higher plants can be affected by the development of the maternal tissue (Li et al., 2011) or promoting cell proliferation in the integuments of ovules (Adamski et al., 2009). Our finding that transgenic tomato plants constitutively expressing *VvYABBY4* formed smaller seeds compared with wild-type suggests that *VvYABBY4* may mainly function to restrict endosperm cell expansion during seed growth.

In conclusion, seven grape YABBY genes were analyzed systematically in terms of evolution and expression pattern during ovule development. Functional characterization suggested that *VvYABBY4* may play a role in growth of endosperm cells during seed development, which may provide new insights on genetic pathways involving YABBY genes as potential candidates for seedless grape breeding.

## Data Availability Statement

All datasets for this study are included in the manuscript/**Supplementary Files**.

## Author Contributions

XW and SZ designed the study. SZ and XS contributed to most experiments. YL and JY assisted with the analysis of the results. XW and LW provided guidance on the study. SN modified the language of the manuscript. SZ and XW wrote the manuscript. All of the authors approved the final version of this manuscript.

## Funding

This work was supported by the Joint Funds of National Natural Science Foundation of China (U1603234) and the Program for Innovative Research Team of Grape Germplasm Resources and Breeding (2013KCT-25).

## Conflict of Interest

The authors declare that the research was conducted in the absence of any commercial or financial relationships that could be construed as a potential conflict of interest.
