# Supplementary material for: Genome-Wide Analysis of the YABBY Gene Family in Grapevine and Functional Characterization of VvYABBY4
Source: Front Plant Sci. 2019 Oct 8;10:1207. doi: 10.3389/fpls.2019.01207 (PMC6791920; doi:10.3389/fpls.2019.01207)

**Supplementary Table 1.** The primer sequences used for qRT-PCR amplification.

| **Gene name** | **Forward primer (5'->3')** | **Reverse prime (5'->3')** |
| --- | --- | --- |
| ***VvYABBY1*** | ACACTGTTCTTGCGGTTG | GCTCGCTGGATGTTGAT |
| ***VvYABBY2*** | TACAATGCGGTTTCTGC | TTGGCTCATCCTGGCT |
| ***VvYABBY3*** | CCGAGCAGCTCTGTTATGTC | ATCCACGAAGGTTTACAGGC |
| ***VvYABBY4*** | AGGTAAGGAGGAAATCCAAAGG | AACTAGCTGCCACTCACAAGGT |
| ***VvYABBY5*** | ATTTTAGCGGTGAGTGTCC | AACCTGTTGTATGCTGATGG |
| ***VvYABBY6*** | GTGACCGTCCGATGCG | GAATGCTTCCCTGTGGCT |
| ***VvYABBY7*** | GGACTGCTTCTTCCTGCT | GGTTTCGGTATCTCATCG |
| ***VvACTIN*** | GATTCTGGTGATGGTGTGAGT | GACAATTTCCCGTTCAGCAGT |
| ***VvEF1-α*** | AGGAGGCAGCCAACTTCACC | CAAACCCTGCATCACCAT TC |

**Supplementary Table 2.** The primer sequences used for cloning of grape YABBY genes.

| **Gene name** | **Forward and reverse primer (5'->3')** | **Restriction sites** |
| --- | --- | --- |
| ***VvYABBY4*** | F: GGGGTACCCC ATGTCACTGGACATCACCCCCGAAC | KpnⅠ |
|  | R: GCTCTAGAGC TTAATAGAATCCATGAGCCTTGTGT | XbaⅠ |

Note：The underline indicate the restriction sites.

**Supplementary Table 3.** Synteny blocks of YABBY genes within grape genome.

| **ID** | **region 1 (Grape)** | | | **region 2 (Grape)** | | | **gene in the synteny region** | |
| --- | --- | --- | --- | --- | --- | --- | --- | --- |
|  | **Chr** | **Start** | **Stop** | **Chr** | **Start** | **Stop** | **gene 1** | **gene 2** |
| 82 | chr2 | 4287650 | 5261179 | chr15 | 15476735 | 14343591 | *VvYABBY3* | *VvYABBY7* |
| 125 | chr8 | 115793 | 7227039 | chr6 | 14796395 | 9415972 | *VvYABBY5* | *VvYABBY4* |

**Supplementary Table 4.** Synteny blocks of YABBY genes between grape and *Arabidopsis* genomes.

| **ID** | **region 1 (Ath)** | | | **region 2 (Grape)** | | | **gene in the synteny region** | |
| --- | --- | --- | --- | --- | --- | --- | --- | --- |
|  | **Chr** | **Start** | **Stop** | **Chr** | **Start** | **Stop** | **gene 1** | **gene 2** |
| 0 | chr1 | 8295185 | 8404301 | chr1 | 7494007 | 8210725 | *AtINO* | *VvYABBY2* |
| 1 | chr1 | 25964468 | 26052567 | chr1 | 48574 | 526024 | *AtCRC* | *VvYABBY1* |

**Supplementary Table 5. The expression of YABBY family genes at three different periods in** **seedless and seedless grapes according to the published transcriptome data (Wang et al., 2016).**

|  |  | **stage1** | |  |  | **stage2** | |  |  | **stage3** | |  |  |
| --- | --- | --- | --- | --- | --- | --- | --- | --- | --- | --- | --- | --- | --- |
|  |  | **seedless** | **seeded** | **ratio** | **P-value** | **seedless** | **seeded** | **ratio** | **P-value** | **seedless** | **seeded** | **ratio** | **P-value** |
| **GSVIVG01012246001** | ***VvYABBY1*** | 0.00 | 0.00 |  | NA | 0.00 | 0.00 |  | NA | 0.00 | 0.00 |  | NA |
| **GSVIVG01013778001** | ***VvYABBY2*** | 0.00 | 0.00 |  | NA | 0.00 | 0.00 |  | NA | 0.00 | 0.00 |  | NA |
| **GSVIVG01001269001** | ***VvYABBY3*** | 3.39 | 0.06 | 56.50 | 0.00 | 1.93 | 0.14 | 13.79 | 0.00 | 0.89 | 0.35 | 2.54 | 0.83 |
| **GSVIVG01037533001** | ***VvYABBY4*** | 4.47 | 0.23 | 19.43 | 0.00 | 1.84 | 0.12 | 15.33 | 0.00 | 0.43 | 0.00 | ! | 0.60 |
| **GSVIVG01022586001** | ***VvYABBY5*** | 10.06 | 0.39 | 25.79 | 0.00 | 5.13 | 0.32 | 16.03 | 0.00 | 2.83 | 0.54 | 5.24 | 0.16 |
| **GSVIVG01015567001** | ***VvYABBY6*** | 0.10 | 0.04 | 2.50 | 1.00 | 0.05 | 0.10 | 0.50 | 1.00 | 0.04 | 0.15 | 0.27 | 0.83 |
| **GSVIVG01027648001** | ***VvYABBY7*** | 4.17 | 0.24 | 17.38 | 0.00 | 2.86 | 0.09 | 31.78 | 0.00 | 0.25 | 0.10 | 2.50 | 1.00 |

Note: Gray plaid represents significant differences

**
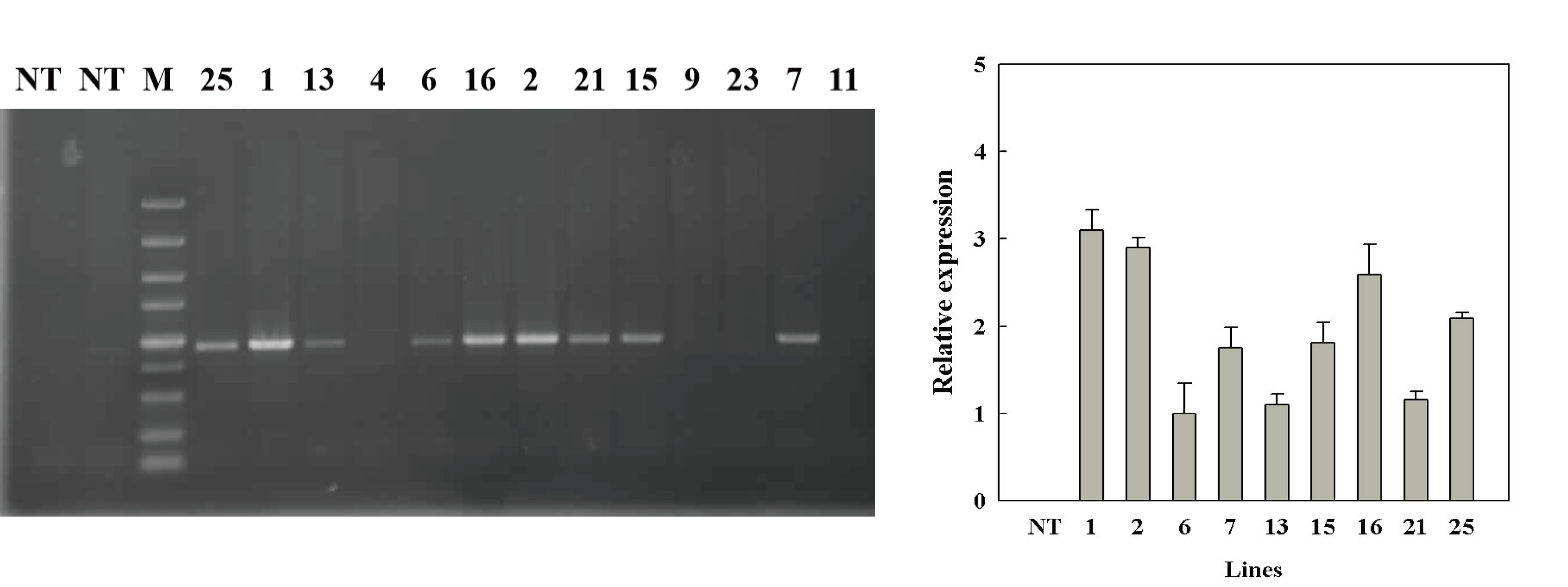
Supplementary Figure 1. Identification and expression level of *VvYAB4* in transgenic plants. (A)** PCR verification of the insertion of recombinant plasmid in trasgenic plants. NT: notransgenic plants M: DL5000. **(B)** Expression level of *VvYAB4* in transgenic plants.

**A**
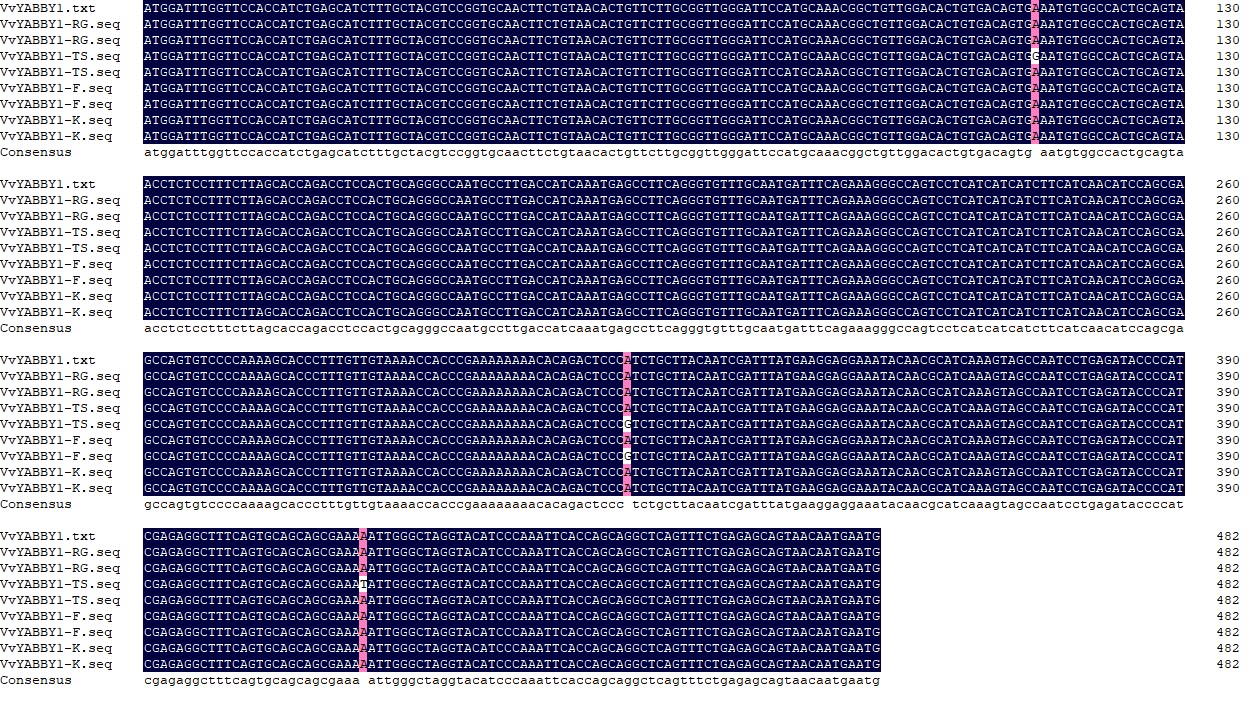


**B**
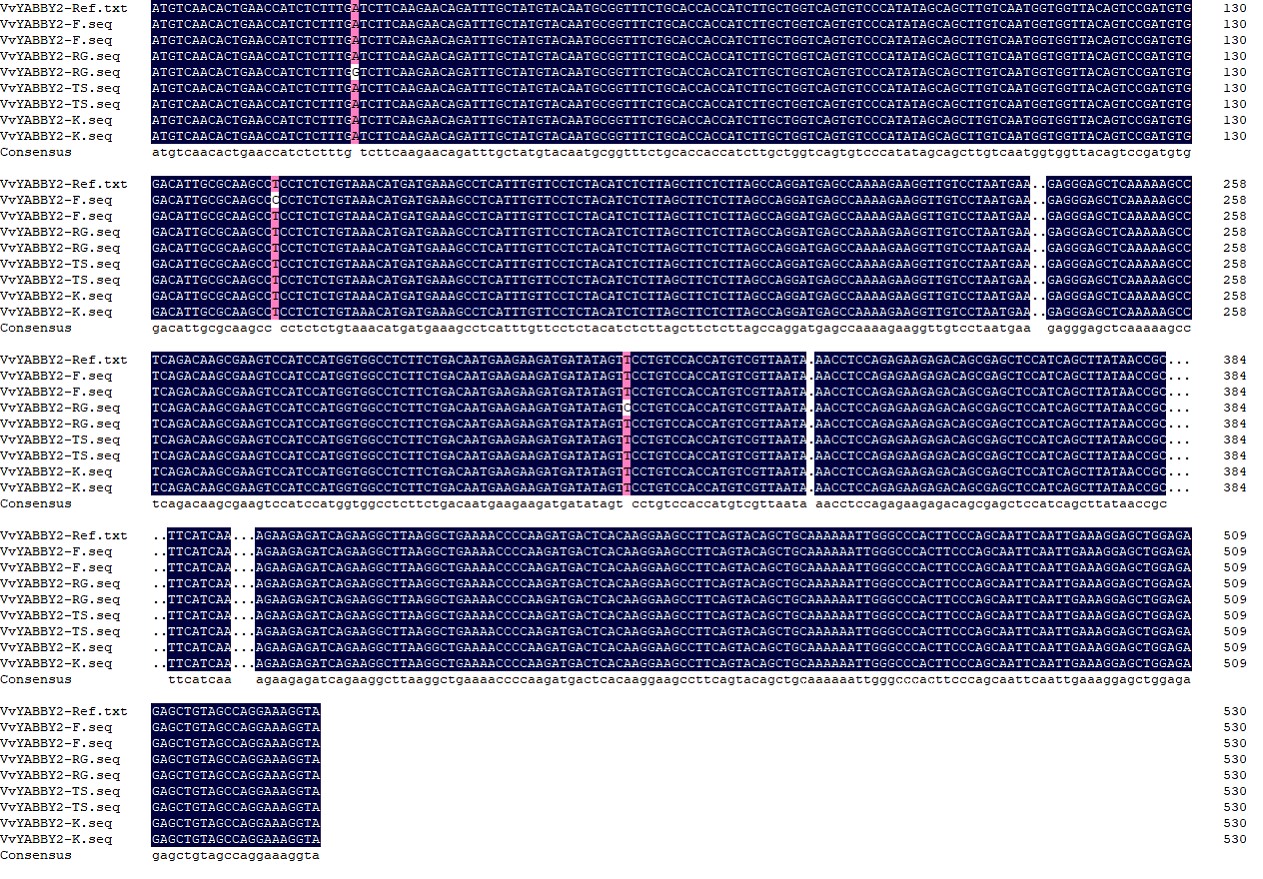


**C**
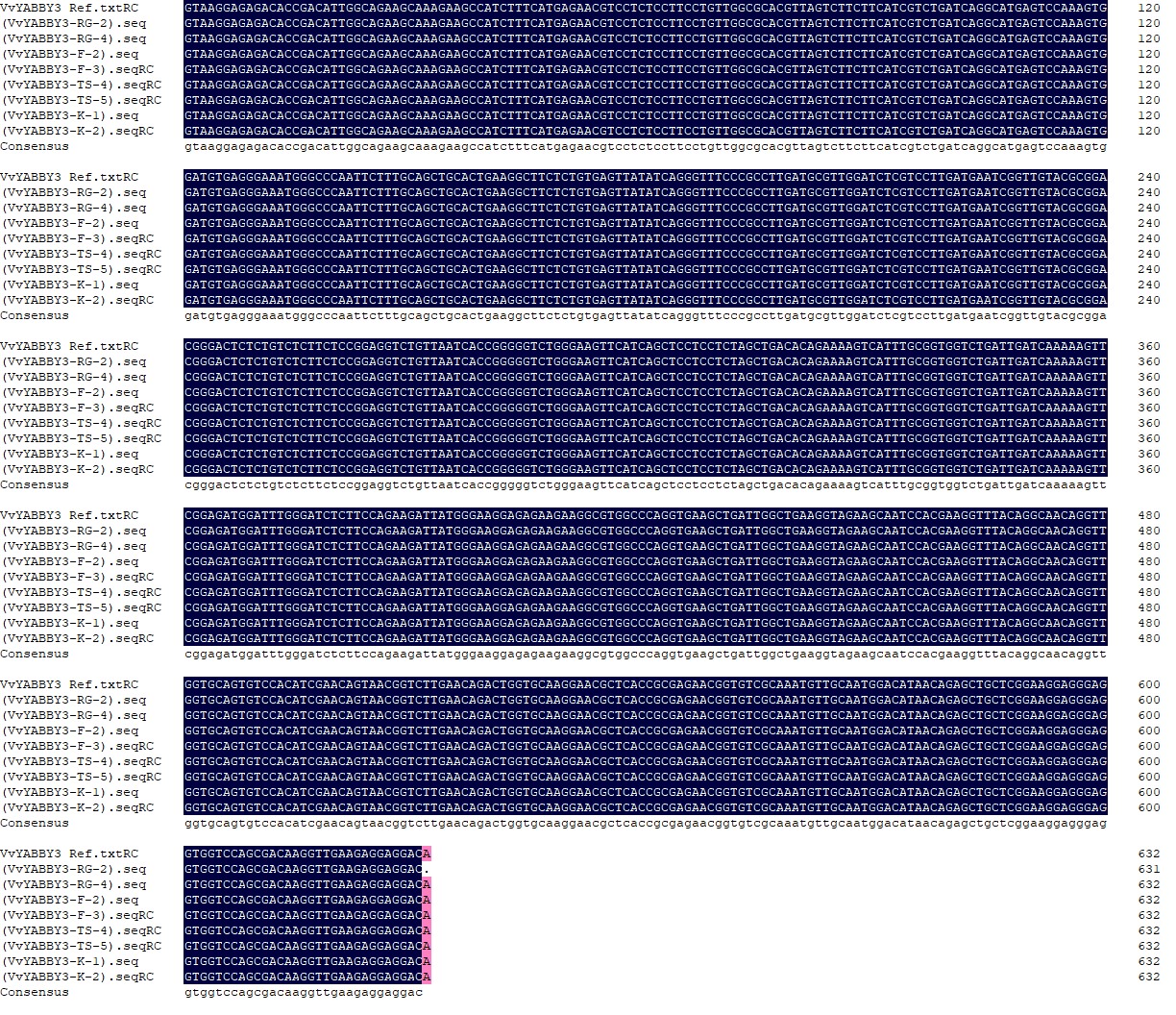


**D
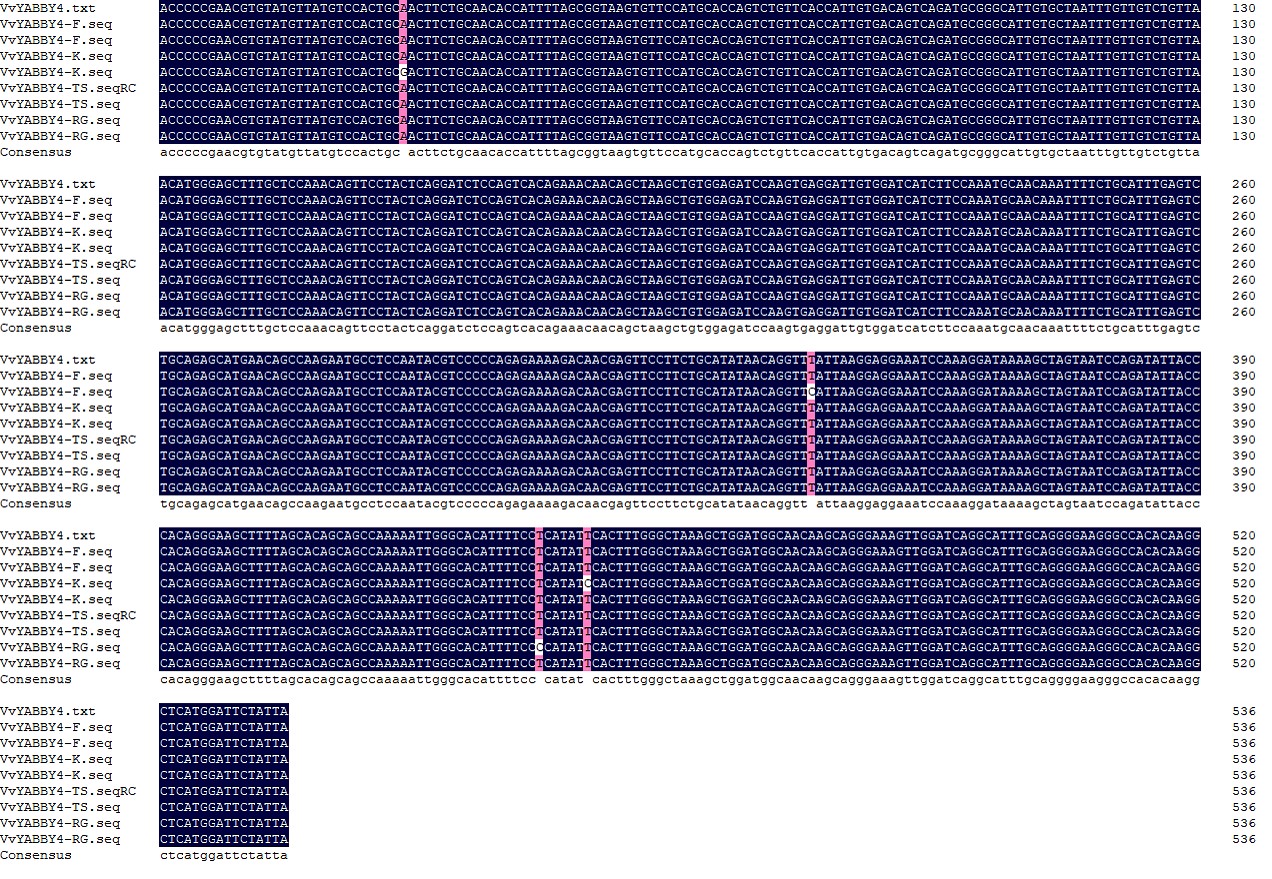
E
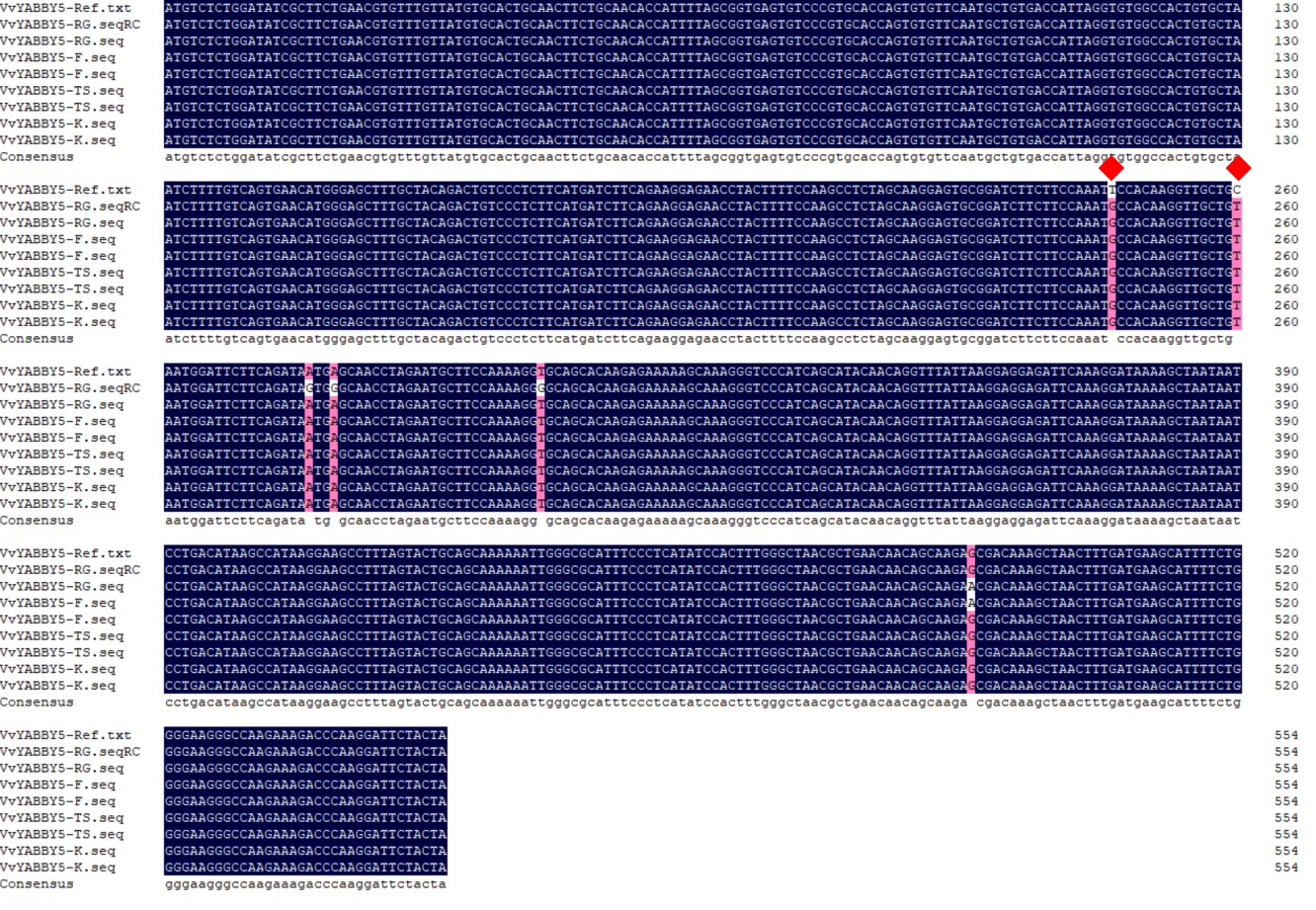
F
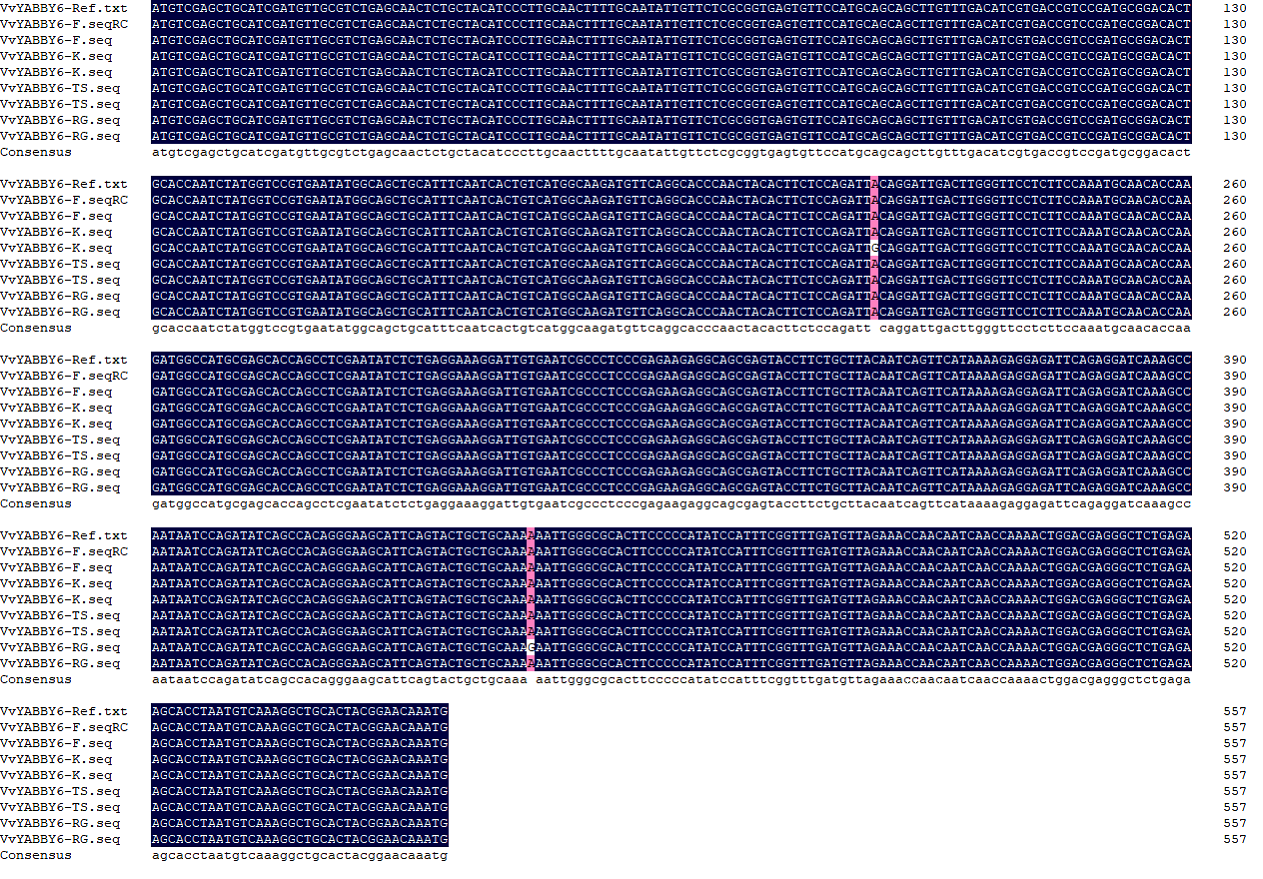
**

**G
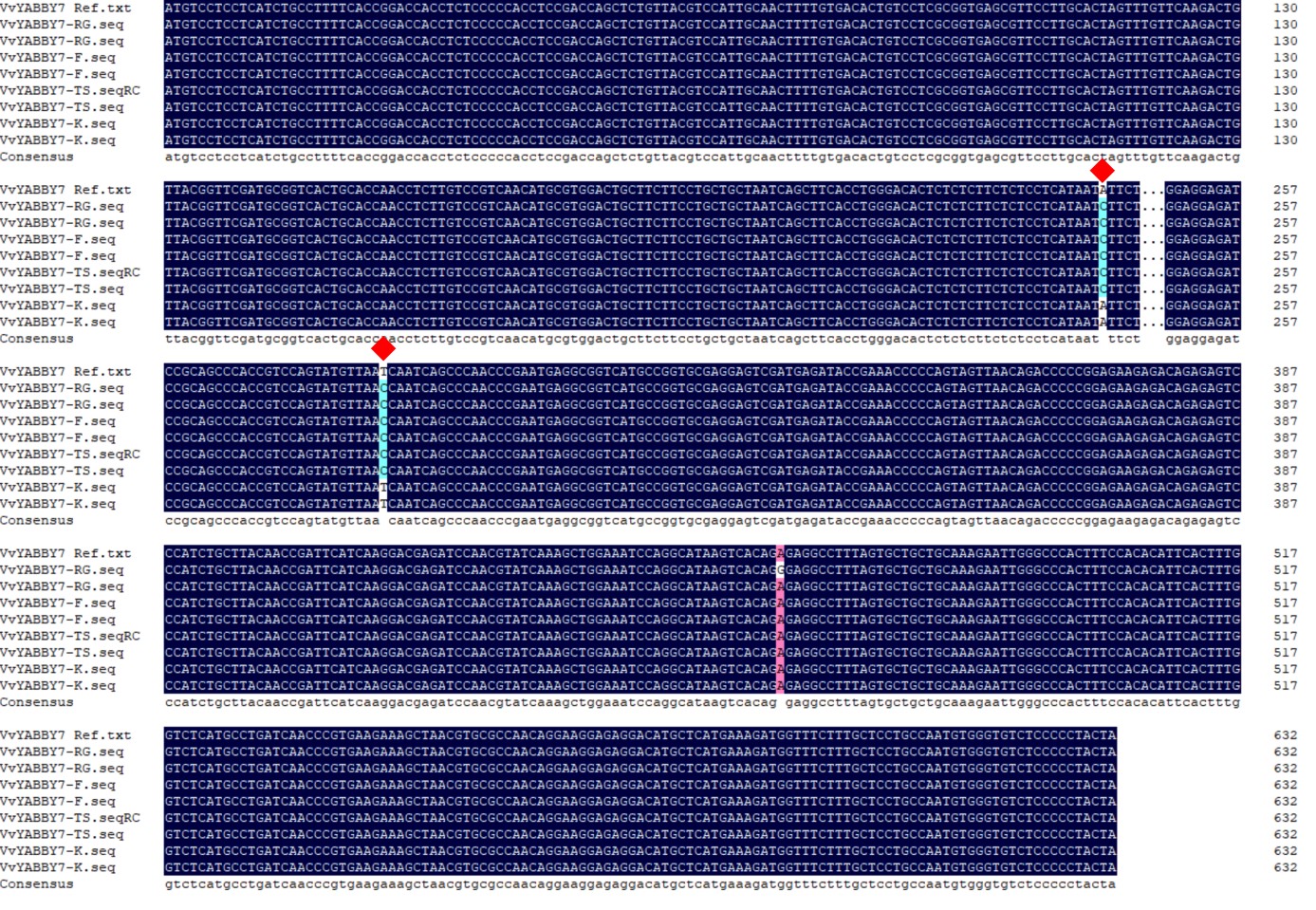
Supplementary Figure 2. Gene sequences alignment of grape YABBY family in four grape varieties. (A)-(G)** represent *VvYABBY1- VvYABBY7*, respectively.

The red mark indicates the difference point.


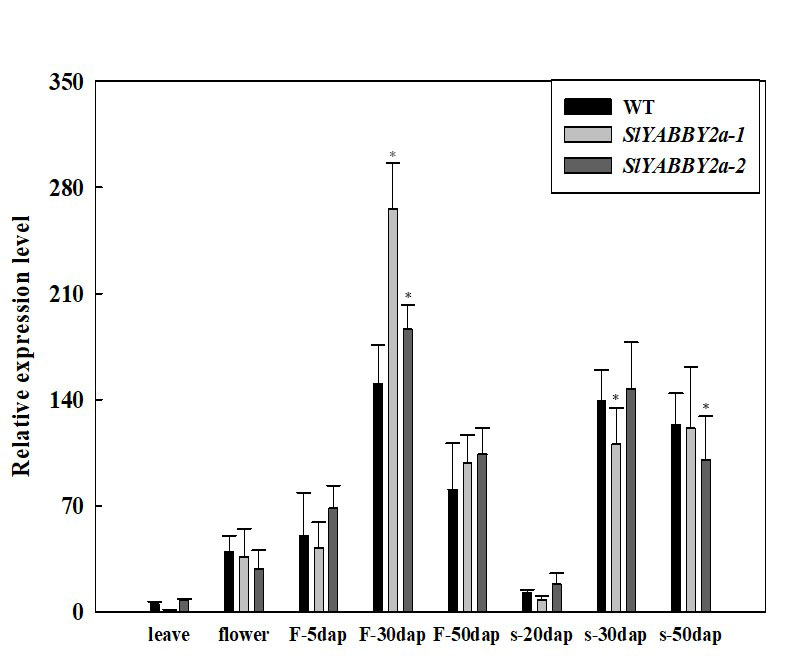

Supplement: Supplementary file 1 [file Table_1.doc]
